# Gaussian Accelerated Molecular Dynamics Simulations Investigation on the Mechanism of Angiotensin-Converting Enzyme (ACE) C-Domain Inhibition by Dipeptides

**DOI:** 10.3390/foods11030327

**Published:** 2022-01-25

**Authors:** Congcong Li, Kaifeng Liu, Siao Chen, Lu Han, Weiwei Han

**Affiliations:** Key Laboratory for Molecular Enzymology and Engineering of Ministry of Education, School of Life Science, Jilin University, Changchun 130012, China; congcong17@mails.jlu.edu.cn (C.L.); liukf1220@mails.jlu.edu.cn (K.L.); sachen20@mails.jlu.edu.cn (S.C.); luhan@jlu.edu.cn (L.H.)

**Keywords:** angiotensin-converting enzyme (ACE), inhibitory peptides, molecular mechanism, Gaussian accelerated molecular dynamics (GaMD) simulations

## Abstract

Angiotensin-converting enzyme (ACE)-inhibitory peptides extracted from food proteins can lower blood pressure by inhibiting ACE activity. A recent study showed that the inhibitory activity of IY (Ile-Tyr, a dipeptide derived from soybean protein) against ACE was much higher than that of LL (Leu-Leu), although they had similar hydrophobic and predicted activity values. It was difficult to reveal the deep molecular mechanism underlying this phenomenon by traditional experimental methods. The Apo and two complex systems (i.e., ACE–LL and ACE–IY) were therefore subjected to 1 μs long Gaussian accelerated molecular dynamics (GaMD) simulations. The results showed that the binding of IY can cause obvious contraction of the active site of ACE, mainly manifested by a significant lateral shift of α13, α14, and α15. In addition, hinge 2 and hinge 3 were more stable in the ACE–IY system, while these phenomena were not present in the ACE–LL system. Moreover, the α10 of the IY-bound ACE kept an inward state during the simulation progress, which facilitated the ACE to remain closed. However, for the LL-bound ACE, the α10 switched between two outward states. To sum up, our study provides detailed insights into inhibitor-induced conformational changes in ACE that may help in the design of specific inhibitors targeting ACE for the treatment of hypertension.

## 1. Introduction

Hypertension is an epidemic cardiovascular disease that has developed into a serious global public health problem [1,2]. Angiotensin-converting enzyme (ACE) is a critical enzyme involved in the renin–angiotensin–aldosterone system (RAAS) and the kallikrein–kinin system (KKS) [3,4], which play an essential part in modulating blood pressure and cardiovascular fitness. Inhibition of ACE activity is considered to be an effective treatment for hypertension. Therefore, ACE is regarded as a suitable target for antihypertensive drug development. The human body contains two isomers of ACE [5]: (1) a somatic cell type (sACE), which has two active sites (N- and C-terminal) and can be generated by a variety of tissues; (2) a testicular form (tACE), which contains only the C-terminal active site and presents only in spermatogenic cells [6].

Although the widely utilized synthetic ACE inhibitors, such as captopril, lisinopril, and enalaprilat, are currently the mainstay in the treatment for hypertension, they can also produce a serious variety of side effects such as dry cough, renal dysfunction, and angioedema [7,8,9]. This has drawn more attention to novel therapeutic agents. At present, the extensively used ACE inhibitors are all peptide analogues [10]. As a result, food-derived ACE-inhibitory peptides have attracted increasing attention due to the fact of their milder side effects during treatment. Currently, ACE-inhibitory peptides can be obtained from dairy products [11,12], eggs [13], seafood [14,15,16], and plants [17,18,19]. Moreover, a number of ACE-inhibiting peptides derived from natural food materials [20,21] have been successfully developed as effective alternatives to synthetic drugs for the prevention of hypertension in a safer manner. Experimental studies on ACE-inhibitory peptides have mainly focused on preparation, purification, and identification [22,23,24], but theoretical studies are less frequent. Unfortunately, these conventional experimental methods cannot reveal the mechanisms at the atomic level by which peptides exert their inhibitory effects. Thus, direct information on the interaction between ACE-inhibitory peptides and ACE is very limited. Computational molecular modeling techniques can rapidly and efficiently provide atomic-level mechanisms for the interaction between ligands and receptors [25,26,27,28].

A recent study by Xu et al. [29] showed that IY was a potent ACE inhibitor that can be obtained from soybean isolate protein (SPI). The experimental results demonstrated that the inhibitory activity of IY was 93.30%, and the IC_50_ value was 0.53 ± 0.02 μM. However, LL, also as a dipeptide, showed only 1.38% inhibition activity; more importantly, its hydrophobicity value, predicted activity score, and amino acid composition were similar to those of IY. It can be speculated from the above results that in addition to these three factors affecting the effect of ACE inhibition, other factors, such as the spatial conformation of the peptide and the conformational changes of ACE caused by ACE-inhibitory peptides binding, were probably significant contributing factors.

In this study, 1 μs GaMD simulations [30] were performed after conventional molecular dynamics (cMD) simulations for Apo (ligand-free ACE) and LL-/IY-bound ACE complexes to elucidate the molecular mechanisms underlying the different inhibitory activities of the two dipeptides. GaMD is able to explore the conformational space of biological macromolecules without the setting of predetermined reaction coordinates. This unconstrained enhanced sampling method has successfully been applied to investigate conformational changes of proteins [31,32,33,34], protein folding [35], protein–ligand binding [36], membrane proteins [37], etc. Thus, GaMD is suitable for studying proteins such as ACE. Based on the obtained trajectories, we first investigated the effect of the spatial conformation of the dipeptide on its binding to ACE. Afterwards, the effect of inhibiting peptide binding on protein conformation was also studied, suggesting that IY or LL binding resulted in a noticeable difference in the conformational change of ACE. This difference, in turn, affected the binding of the inhibitor to the protein. This study may provide a basis for the rational design of peptide inhibitors for ACE.

## 2. Methods

### 2.1. Simulation System Preparation

**Figure 1:**
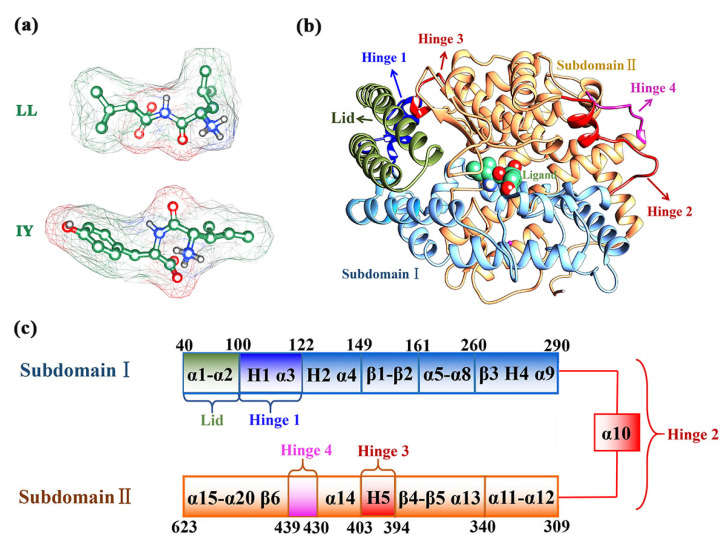
Stereo structures of the ligands and complex: (**a**) 3D structures of IY and LL. IY is the dipeptide Ile-Tyr, and LL is the dipeptide Leu-Leu; (**b**) complex of the ACE (angiotensin-converting enzyme) and inhibitory peptide; (**c**) detailed diagram of the secondary structure of ACE.

The 3D structures of LL and IY were modeled using Discovery Studio Visualizer v21.1 [38], and the structures are shown in Figure 1a. Next, we used Gaussian 09 [39] to optimize the structures of the two dipeptides at the level of B3LYP/6-31G* to obtain the optimal conformation for subsequent molecular docking. Molecular docking for different protein receptors of ACE with ACE-inhibitory peptides in the database showed that IY had the highest affinity with the tACE (PDB ID:2OC2) [29]. Thus, we downloaded the structure of tACE–ligand complex directly from the RCSB database (https://www.rcsb.org/, accessed on 22 May 2007) (PDB code: 2OC2) [40]. The ligand of the obtained complex was removed using DS, and the remaining part was the Apo system. The detailed secondary structure composition of ACE is shown in Figure 1c. LL and IY were docked to the active site of the Apo protein with Autodock 4.2 [41] to form the ACE–LL, and ACE–IY complexes, respectively (Figure 1b). The size of the docking box was set to x = 40, y = 40, and z = 45 and the length of each grid was 0. 0375 nm. The Lamarckian genetic algorithm (LGA) was used to calculate the molecular docking, and the lowest energy structure was selected from the most clustered class of docking results as the initial structures for GaMD simulations.

Systems under study were designated as follows: LL (Leu-Leu) for ACE–LL, IY (Ile-Tyr) for ACE–IY, and Apo for ACE without ligand and used in Section 3.

### 2.2. Equilibrium Simulations

The pmemd.cuda module of AMBER 16 [42] was used to perform conventional MD simulations for three model systems. Prior to the simulation, the Leap module embedded in AMBER was used to generate force field parameters for proteins and dipeptides, both using the ff14SB force field [43]. Afterwards, each system was dissolved in an octahedral box using the TIP3P [44] water model. The distance between the solute surface and the box was set to 10 Å. To prevent edge effects, periodic boundary conditions (PBCs) were applied to the three systems. Appropriate amounts of the antagonistic ions (Cl^−^) were added to neutralize the system. All bonds involving hydrogen atoms were constrained using the SHAKE algorithm [45]. The particle mesh Ewald (PME) algorithm [46] was used to handle non-bonded electrostatic interactions, and the cut off was 10 Å. Before the production simulation, energy minimization was executed for the three systems to eliminate atomic collisions in the initial structure. In the minimization phase, the steepest descent algorithm and conjugate gradient algorithm were performed for 5000 steps each. Then, the three models were gradually heated to 300 K under NVT ensemble. Finally, 50 ns simulations were carried out for the equilibrium of the systems under the NPT ensemble. The entire simulation used a time step of 2 fs.

### 2.3. Gaussian Accelerated Molecular Dynamics Simulations

The initial structures used by the GaMD simulations were obtained from the well-balanced structure of the cMD simulations. In the case of the GaMD approach, the harmonic boost potential was added so that the energy barrier could be reduced by smoothing the potential surface and, thus, accelerating the transition between different conformational states for the purpose of enhanced sampling [30]. Here, the increased lifting potential followed the Gaussian distribution, allowing the original potential surface to be easily recovered. In addition, GaMD has the benefit of not requiring any predetermined reaction coordinates or collective variables (CVs). Thus, this enhanced simulation approach is very suitable for studying the dynamics of complex biological systems. 

In this study, we applied the dual potential boost to the GaMD simulations. The dual potential boost parameters were defined by the previous 50 ns cMD simulations. Afterwards, a 50 ns GaMD simulation was carried out. Lastly, 1 μs GaMD simulations were conducted in the NVT ensemble with coordinates saved every 10 ps.

### 2.4. Trajectory Analysis

All analyses, which included RMSD, RMSF, *R_g_*, SASA, and DCCM, were computed using Amber16’s Cpptraj module [47]. Principal component analysis (PCA) [48] was also calculated using Cpptraj. It is a widely used dimensionality reduction method that describes the coordinated motion of the entire protein. The free energy landscapes (FELs) are often used to find the dominant conformation and its corresponding potential barrier. Here, we used the PyReweighting scheme developed by the McCammon group [49] to recover the original FEL. The 10th order of the Maclaurin series of expansion was applied to reweight the total boost energy on each trajectory. Reweighted trajectories were used for all analyses.

## 3. Results and Discussion

The dynamics changes in ACE after IY and LL binding were investigated by implementing 1 μs GaMD simulations. In addition, the two complexes were compared with Apo to elucidate the effect of inhibitor binding on the structure dynamics of ACE.

### 3.1. Structural Stability and Flexibility of the ACE–Inhibitor Complex

**Figure 2:**
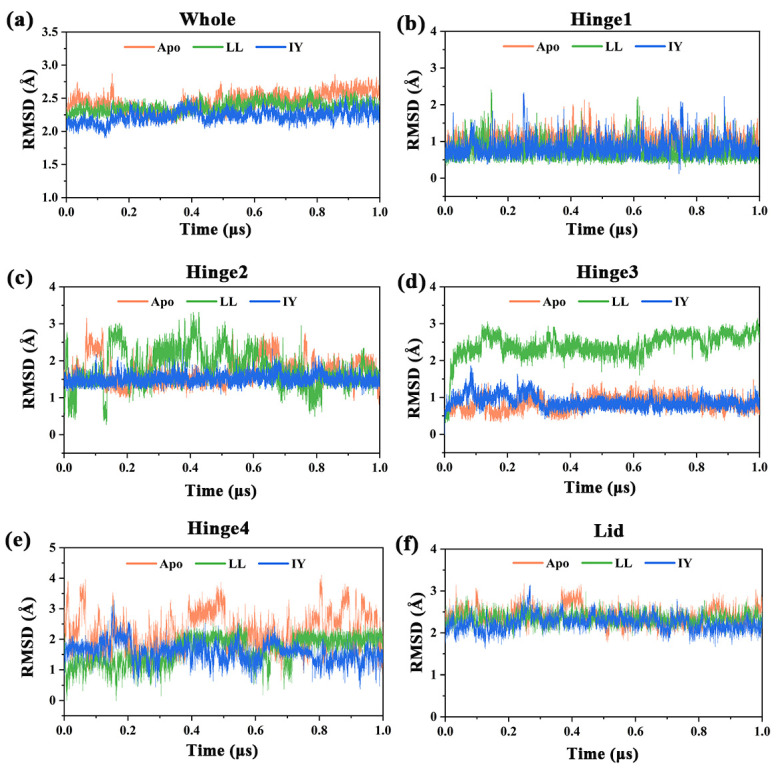
The temporal evolution of the RMSDs from their initial structure of three complexes in the region of (**a**) the whole protein, (**b**) hinge 1, (**c**) hinge 2, (**d**) hinge 3, (**e**) hinge 4 and (**f**) the lid. RMSD is root mean square deviation.

The stability and convergence of the simulated systems were assessed based on the root mean square deviation (RMSD) of protein backbone atoms with respect to the initial structure (Figure 2). It can obviously be seen from Figure 2a that the three systems basically reached equilibrium after 0.42 μs. Although both the Apo and peptide-bound systems kept relatively stable RMSD fluctuations throughout the 1 μs simulation, the average RMSDs of the LL-bound or IY-bound ACE decreased by ~0.1 and ~0.2 Å, respectively, compared with that of the Apo (Table 1). This implied that the binding of inhibitory peptides, particularly the IY peptide, led to significant structural variations in the protein relative to the Apo. In addition, RMSDs of several important regions of the enzyme were calculated to study the structural changes in these areas, which included hinge 1, hinge 2, hinge 3, hinge 4, and the lid. As shown in Figure 2b–f, in the case of ACE–IY, the deviations in the hinge 2 and hinge 3 regions were significantly smaller than the other two systems. In the ACE–IY system, the average RMSDs for hinge 2 and hinge 3 were 1.50 and 0.88 Å, respectively, compared with 1.8 and 2.43 Å in the ACE–LL system (Table 1). In contrast, for hinge 4 and the lid regions, the differences in the mean RMSD values between ACE–IY and ACE–LL were small, with only the ACE–IY system exhibiting smaller RMSD values than the ACE–LL system after 0.8 μs. A similar deviation trend was observed for the three systems in hinge 1. In general, RMSD analysis indicated that ACE combined with IY was more stable compared to ACE–LL, especially at hinge 2 and hinge 3. 

**Figure 3:**
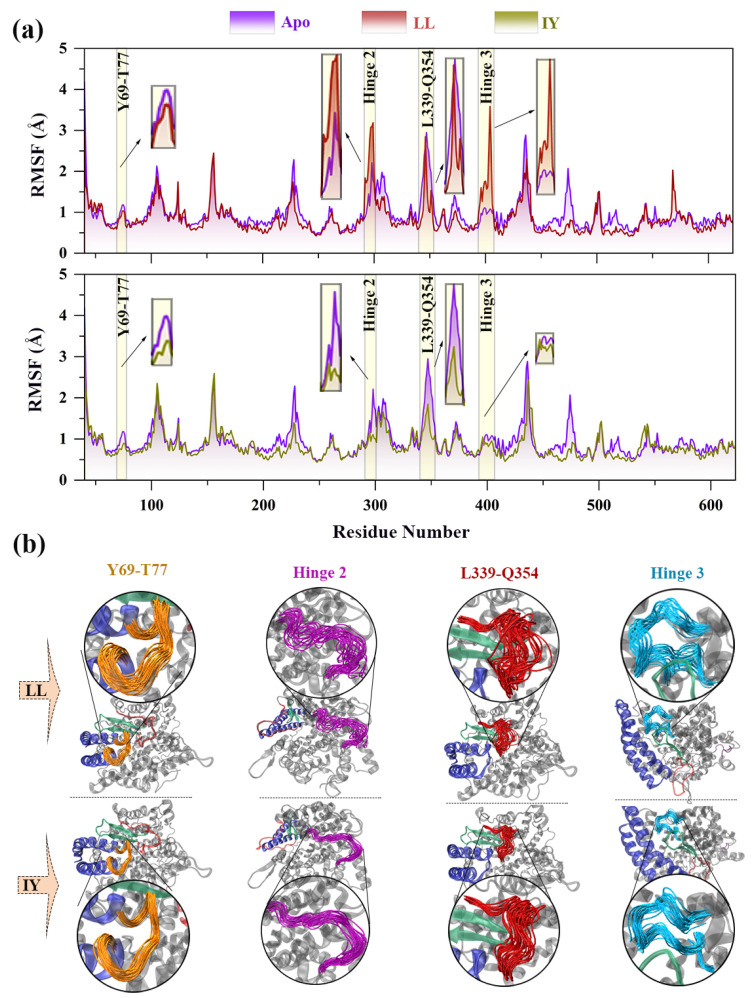
(**a**) RMSFs of Cα atoms in the three systems, and critical regions are highlighted with rectangles. RMSF is root mean square fluctuation; (**b**) superposition of extracted protein conformations from MD (molecular dynamics) trajectories for critical regions. The Apo means ligand-free protein, LL represents the complex system of protein and dipeptide Leu-Leu, and IY stands for complex system of protein and dipeptide Ile-Tyr.

Subsequently, root mean square fluctuation (RMSF) values of C_α_ atoms were calculated to assess the flexibility of each residue upon binding to the inhibitor and compared to the corresponding Apo system (Figure 3). It is evident from Figure 3 that the fluctuation of residues in the ACE–LL complex was higher than that of ACE–IY. This was consistent with the result that the LL-bound ACE had an overall higher RMSD than the ACE–IY system. As seen in Figure 3a, the large deviations were mainly caused by hinge 2 and hinge 3. The fluctuations in these two hinge regions were much higher for ACE–LL than other systems. Apart from that, loops between lid’s α1–α2 (residues 69–77), and α12–β4 (residues 339–354) also showed slight differences in volatility, with slightly higher volatility in the ACE–LL system than in the ACE–IY system, while they were both lower than in the Apo system. Figure 3b displayed a comparison of the conformations sampled by ACE–LL and ACE–IY in the GaMD simulations, which suggested that the four regions mentioned above had higher mobility in ACE–LL than in ACE–IY, which was supported by the RMSF calculations. These indicated that the residues 69–77, hinge 2, residues 339–354, and hinge 3 were stabilized upon binding to the IY inhibitor, especially hinge 2 and hinge 3. Notably, the RMSF variation trend of ACE bound to IY was the same as that already reported for ACE bound to lisinopril [50], which was one of the most widely used inhibitors [4]. This suggested that IY and lisinopril may have similar effects on the conformational changes of ACE during molecular dynamics simulations. It also indicated that IY had a good inhibitory effect on ACE.

**Figure 4:**
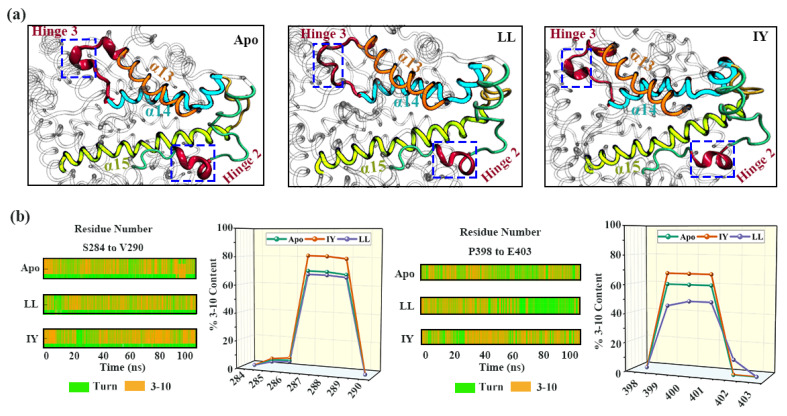
Analysis of the secondary structural changes of proteins: (**a**) representative snapshots of the three systems, and hinge 2 and hinge 3 are highlighted with rectangles; (**b**) DSSP (dictionary of secondary structure of protein) results for the three complexes and the helix probabilities of the corresponding residues. The Apo means ligand-free protein, LL represents the complex system of protein and dipeptide Leu-Leu, and IY stands for complex system of protein and dipeptide Ile-Tyr.

Next, we investigated the helical content of the α9 (in hinge 2) and H5 (in hinge 3) throughout the simulation shown in Figure 4. It the distortion of α9 and H5 in ACE was obviously displayed when it was bound with LL. On the other hand, in the case of IY, bound states and no such severe distortion was observed (Figure 4a). The probability of the helical content of α9 and H5 is shown in Figure 4b. The results of the protein’s secondary structural changes were consistent with those of RMSF.

### 3.2. Dynamic Cross-Correlation Map 

**Figure 5:**
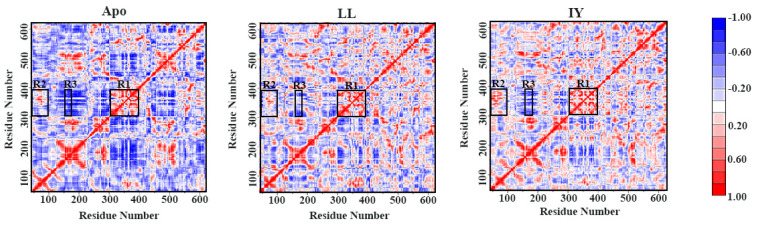
The dynamic cross-correlation map for the 1 μs GaMD simulation trajectories of the three systems. The positive regions are colored in red, whereas the negative regions are shown in blue, representing correlated and anti-correlated motions between residue atoms, respectively. The Apo means ligand-free protein, LL represents the complex system of protein and dipeptide Leu-Leu, and IY stands for complex system of protein and dipeptide Ile-Tyr.

In order to clarify the effect of the inhibitory peptide binding on the movement within the protein chain in both systems, the dynamic cross-correlation matrix (DCCM) was calculated for each residue (Figure 5). Overall, both inhibitor-bound systems exhibited a reduction in anti-correlated movements compared to the Apo system. In the ACE–IY system, the Region R1 showed higher positive correlation movement than the ACE–LL complex and the Apo system, and the R1 region was an important component of the active site. This showed that the binding of the IY inhibitor stabilized the active site of the structure. Region 2 is the lid of the protein, which displayed stronger positively correlated motion in ACE–IY than the other two systems. Region 2 is the protein’s lid, which showed a stronger positive correlation movement in ACE–IY than the other two systems. This indicated that the lid closed towards the active site, which may help to explain why the secondary structure of H5 (in hinge 3) for the ACE–IY system could remain stable (Figure 4). R402 (on H5) could form a stable salt bridge with D52 (in the middle of the lid). Thus, the movement of the lid to the active site could maintain the stability of H5. As shown in Region 3, α5 and Region 1 showed a strong negative correlation movement. α5 was on the opposite site of Region 1. Therefore, their movement in the opposite directions facilitated the closure of the cleft. Overall, the DCCM analysis reflected that the binding of an IY inhibitor stabilized the active site region and enhanced the lateral shift of the lid and α5 for active site cleft closure. 

### 3.3. Analysis of the Interaction between ACE and Dipeptide Inhibitors

**Figure 6:**
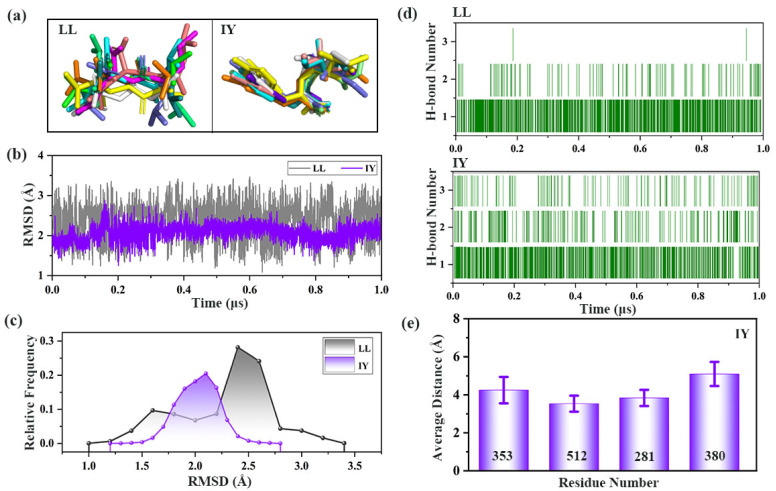
Analysis of the interaction between ACE (angiotensin-converting enzyme) and inhibitory peptides: (**a**) ligand poses of 10 superimposed structures over 1 μs for IY and LL. IY is the dipeptide Ile-Tyr, and LL is the dipeptide Leu-Leu; (**b**) time evolution of the RMSDs (root mean square deviations) and (**c**) corresponding frequencies for LL and IY. LL represents the complex system of protein and dipeptide Leu-Leu, and IY stands for complex system of protein and dipeptide Ile-Tyr; (**d**) evolution of the number of hydrogen bonds formed between ACE and peptides during MD (molecular dynamic) simulations; (**e**) average distance between IY and residues interacting with inhibitor.

Ten average ligand poses were obtained from cluster analysis over 1 μs simulations (Figure 6a). The color of the peptides determined by atom type and pose are shown in Figure S1. The RMSDs of the ligands and the corresponding relative frequencies (Figure 6b,c) showed that the RMSD values of IY were mainly at approximately 2.1 Å, while LL varied between 2.3 and 2.6 Å. This suggests that the IY was very stable during the simulation process; however, the stability of LL decreased, and the direction changed.

**Figure 7:**
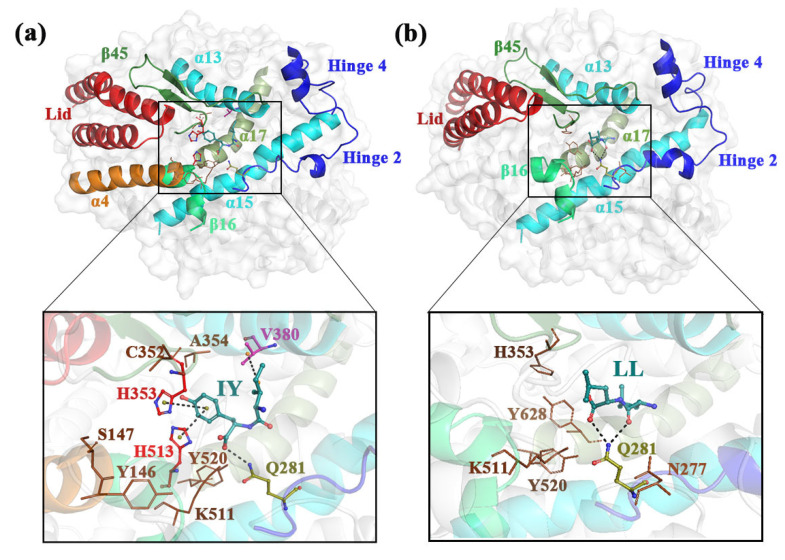
Schematic diagram of bridging of inhibitors across the active site cleft for (**a**) ACE–IY and (**b**) ACE–LL complexes. ACE–IY stands for complex system of protein and dipeptide Ile-Tyr, ACE–LL represents the complex system of protein and dipeptide Leu-Leu.

Representative structures obtained by clustering analysis were used to study detailed protein–inhibitor interactions (Figure 7). As shown in Figure 7a, IY had Pi–Pi stacking interactions with H353 and H513, as well as alkyl hydrophobic interactions with V380. The average distance between these three residues and IY showed that the interaction between these residues and IY was stable (Figure 6e). Some contributions to binding affinity between ACE and inhibitor also came from the hydrogen bonding of the inhibitor to ACE. Residues that had hydrogen bonding interactions with IY included A354, C352, S147, Y146, Y520, K511, and Q281. These interactions synergistically facilitated the bridging of IY across the active site cleft. In comparison, for the ACE–LL system, LL only formed hydrogen bonding interactions with a small number of residues in ACE which were H353, Y628, K511, Y520, N277, and Q281 (Figure 7b). This may help to explain the variation in the secondary structure of α9 in hinge 2 in different systems (Figure 4). Figure 6d also represented that the number of hydrogen bonds formed between IY and ACE were significantly higher compared to LL. This ultimately resulted in suboptimal bridging of LL across the active site cleft (Figure 7b). The above phenomenon explained why IY was more stable than LL in binding to ACE during MD simulations. 

The above analysis showed that most of the residues interacting with IY were the same as those interacting with lisinopril in the crystal structure of the ACE–lisinopril complex found by Natesh et al. [4]. These residues included Y520, K511, H513, and H353, which are important residues for bridging the active cleft. Previously, Jiang et al. [51] found that TFPHGP showed better inhibition of ACE than HWTTQR, and the analysis of the interaction between TFPHGP and ACE indicated that the residues interacting with TFPHGP also included H353, K511, Y520, and H513. In addition, E384 interacted with the inhibitor in both ACE–lisinopril and the ACE–TFPHGP complex system. For ACE–IY, although E384 had no obvious interaction with IY, V380 can complement this role because both E384 and V380 belong to α13, and V380 also played a key role in bridging the active cleft. In contrast, in the ACE–LL system, which lacked H513, A354 and V380 interact with LL. Taken together, the above comparative analysis suggested that, in addition to the effect of simple spatial site resistance, IY can interact with residues that are critical for inhibitory activity and, therefore, exhibit stronger ACE-inhibitory activity than LL.

### 3.4. Structural Characterization of ACE upon Binding LL/IY

#### 3.4.1. IY Inhibitor Binding Leads to ACE Active Site Contraction

**Figure 8:**
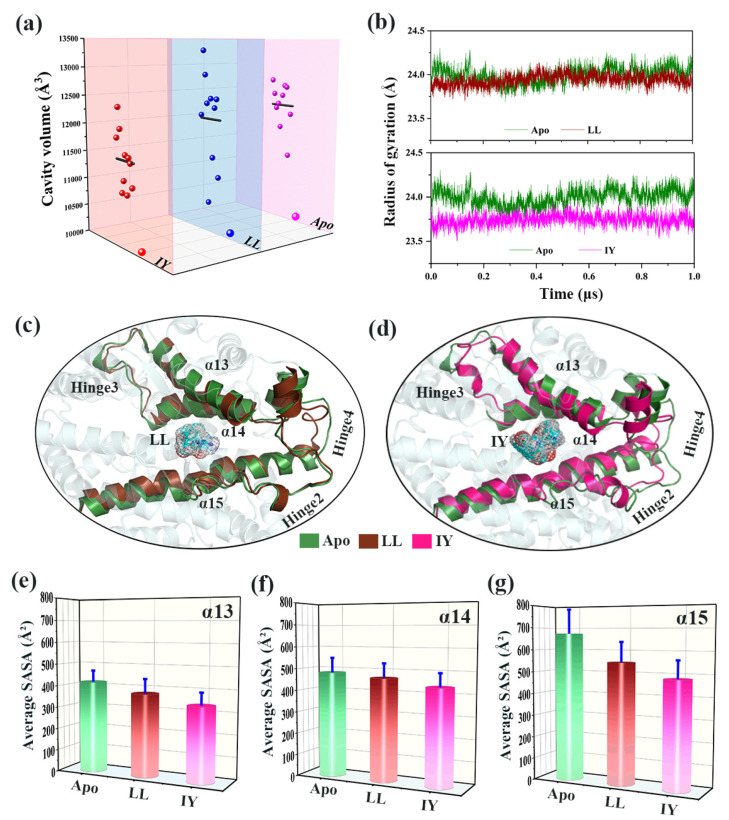
(**a**) Active site volume analysis for the three systems: (**b**) radius of gyration over 1 μs GaMD for the three systems; alignments of (**c**) LL-bound and (**d**) IY-bound representative structures to its apo form; the average SASA of (**e**) α13, (**f**) α14, and (**g**) α15 for the three systems. The Apo means ligand-free protein, LL represents the complex system of protein and dipeptide Leu-Leu, and IY stands for complex system of protein and dipeptide Ile-Tyr.

To investigate whether inhibitory peptide binding had an effect on the conformational change in the active site of ACE, the active site cavity volume of 10 average protein structures obtained from cluster analysis for three systems were calculated using the online server CASTp [52]. The cavity volume of ACE without inhibitor binding was highly dynamic, ranging between 10,949 and 12,387 Å^3^ (Figure 8a). Although the protein was still dynamic upon binding of inhibitory peptide, the cavity volume of the protein bound to IY was substantially decreased. In contrast, the cavity volume for the LL-bound protein was slightly reduced compared to the Apo system.

The radius of gyration (*R_g_*) was calculated to reveal how the tightness of the whole protein changed over time in the three systems (Figure 8b). The *R_g_* for the ACE upon binding IY was smallest in the three systems (i.e., ~23.75 Å), while the other two systems had a similar *R_g_*. This was consistent with the results of cavity volume variations. The representative structure of Apo was aligned to the ACE–LL complex and the ACE–IY complex. The ACE–LL complex aligned well with Apo in most regions of the active site except for the hinge 2 region (Figure 8c). ACE–IY complex was poorly aligned with Apo at α13, α14, α15, hinge 2, hinge 3, and hinge 4 (Figure 8d), suggesting the role for these regions in ligand binding, cleft closure, and complex stabilization.

Next, the SASA of α13, α14, and α15 were calculated for the three systems (Figure S2), and the corresponding average values are shown in the Figure 6e–g. The average SASA values of the ACE–IY complex were relatively small compared to Apo or ACE–LL. The same trend was observed for the three helix regions. Since the inhibitory peptide was hydrophobic, the smaller SASA value indicated that the inhibitor was tightly bound to the active site. Thus, IY bound more tightly to ACE than LL and had the better inhibitory effect. In summary, IY binding caused contraction of the active site which, in turn, facilitated the binding of IY to ACE.

#### 3.4.2. Dynamic Analysis of Active Site

**Figure 9:**
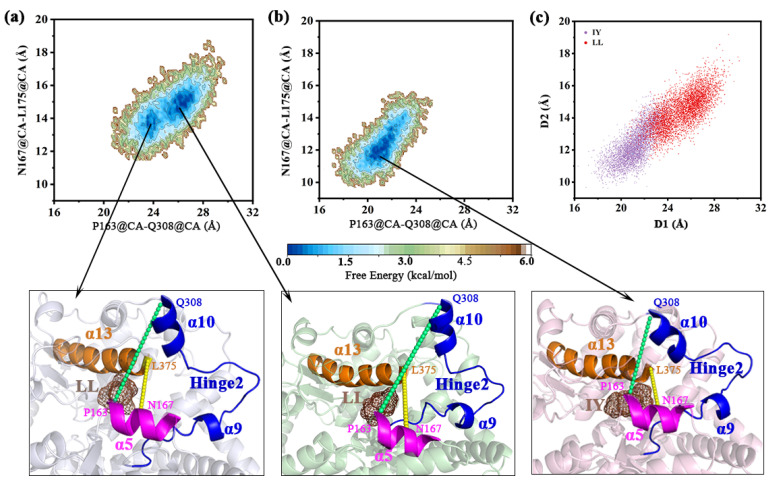
Free energy landscape showing the α10-helix in–out for the complexes (**a**) ACE–LL and (**b**) ACE–IY, ACE–LL represents the complex system of protein and dipeptide Leu-Leu, ACE–IY stands for complex system of protein and dipeptide Ile-Tyr; (**c**) population density of the LL-bound and IY-bound ACE (angiotensin-converting enzyme), D1 was P163@CA-Q308@CA, and D2 was N167@CA-L175@CA. The respective global minimum structures for both structures are also shown below.

As shown in Figure 9, the free energy curve was constructed to investigate the state of the active site in different systems using the distance from Cα of P163 (α5) to Q308 (α10) as the horizontal coordinate and the distance from Cα of N167 (α5) to L375 (α13) as the vertical coordinate. When ACE bound to LL, the free energy curve was characterized by two minimum values (Figure 9a), indicating that the active site was mainly in a fully open or semi-open state. On the other hand, for the energy profile of the ACE–IY system, only a stable global minimum was shown, representing α10-helix-in state, and the α5 moved towards α13 and hinge 2 (Figure 9b). The distance between P163 and Q308 was less than 22 Å, and the distance from N167 to L375 was ~12 Å; the active site was closed at this time. The population density map (Figure 9c) explicitly shows the two distinct states of ACE when binding different inhibitory peptides, suggesting inward movement of the α10 helix and cleft closure in the ACE–IY complex. However, the α10 helix moved outward to varying degrees and the cleft opened in the ACE–LL system.

#### 3.4.3. Motions of Protein via Principal Component Analysis 

**Figure 10:**
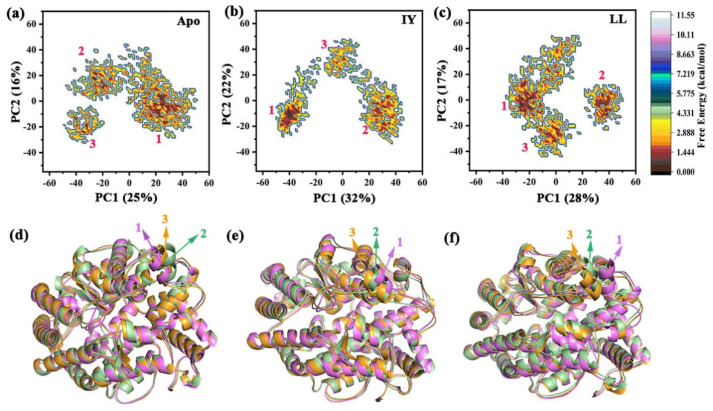
Two-dimensional free energy landscapes (FELs) created by projecting the principal components PC1 (principal component 1) and PC2 (principal component 2) for (**a**) Apo, (**b**) ACE–IY, and (**c**) ACE–LL; the representative structures of (**d**) Apo, (**e**) ACE–IY, (**f**) ACE–LL from cluster analysis are shown in the below panel. Apo means ligand-free protein, ACE–IY stands for complex system of protein and dipeptide Ile-Tyr, ACE–LL represents the complex system of protein and dipeptide Leu-Leu.

Figure 10a–c show the FELs of PC1 and PC2 for Apo as well as for both complexes. The two largest PCs (i.e., PC1 and PC2) accounted for 41%, 54%, and 45% of the overall fluctuations in Apo, ACE–IY, and ACE–LL, respectively. The proportion of PC1 and PC2 in the ACE–IY system were higher than the other two systems, indicating that the IY inhibitor stabilized the dynamic structure of ACE. Among the three systems, the conformational subspace distribution of ACE–IY was different from the other two systems, and the subgroup of ACE–IY was more unitary. This suggested that the conformational space of the ACE–IY complex was smaller than that sampled by Apo and ACE–LL. In addition, to visualize the detailed motion of the proteins, representative structures of subspace were shown in Figure 10d–f. The representative structures of the three different clusters of ACE–IY were conserved, while representative structures of Apo and ACE–LL appear to vary significantly across the three clusters. These again suggested that the binding of IY inhibitors increased the stability of the protein.

#### 3.4.4. The Molecular Mechanism behind the Different States of the Active Site

The S284 (on α9 of hinge 2) was observed to hydrogen bond to the E376 at the tip of α13, while the D377 in α13 formed a hydrogen bond with T372 (on the loop between α13 and β5). However, T372 also interacted with E162 on α5. The α5 thus moved inwards towards α13 and hinge 2, but was repelled by the negative charge from E376, D377, and D288, which resulted in the opening/closing of the active site. Such significant hydrogen bonding interactions that described the differences in the molecular mechanism behind IY and LL binding would explain how different states of the active site vary. 

**Figure 11:**
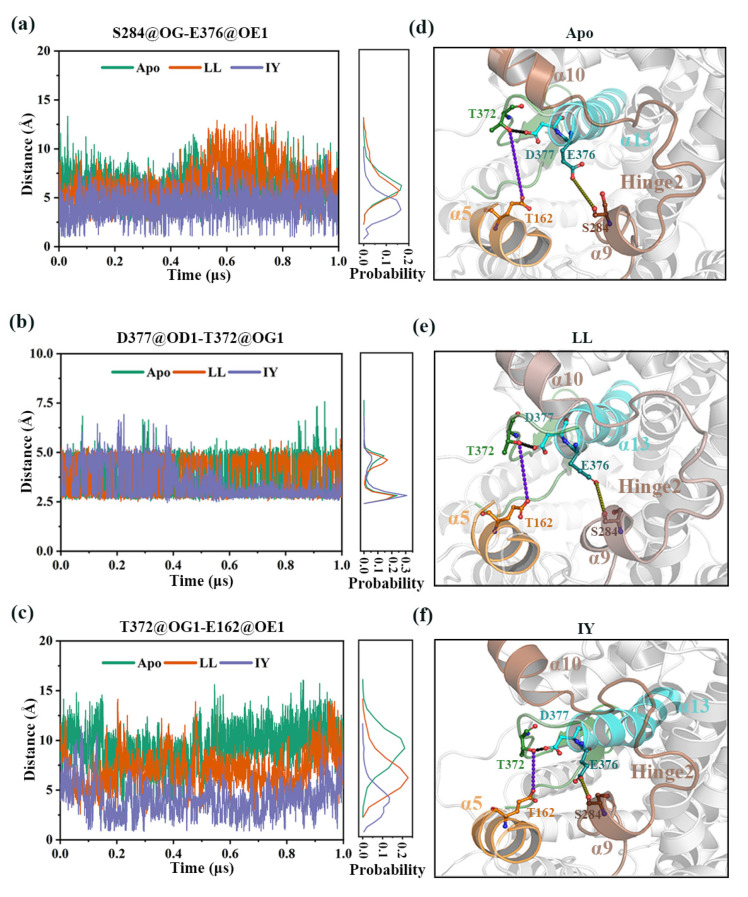
Interactions affecting the active site in ACE upon binding of LL and IY. The hydrogen bonding between different residues, such as (**a**) S284–E376, (**b**) D377–T372, and (**c**) T372–E162, are shown; the schematic diagrams highlighting the critical interactions for (**d**) Apo, (**e**) ACE–LL, and (**f**) ACE–IY. Apo means ligand-free protein, ACE–LL represents the complex system of protein and dipeptide Leu-Leu, ACE–IY stands for complex system of protein and dipeptide Ile-Tyr.

As shown in Figure 11a, the hydrogen bond interaction between S284 and E376 remained stable throughout the simulation of the ACE–IY system, while this interaction fluctuated for the Apo and ACE–LL system. This may be due to the fact that IY can interact with residues in both α9 and α13, thus bringing the distance between the two regions closer. On the other hand, the lack of interaction between LL and α13 led to an unstable distance between S284 and E376. In system ACE–IY, the distance between D377 and T372 was more stable than the other two systems after 0.4 μs (Figure 11b), which may be related to the interaction between IY and V380. The distance between T372 and E162 maintained at ~4 Å. In contrast, the other two systems were difficult to form stable hydrogen bonding interactions (Figure 11c). Altogether, our results deduced that the interaction network between S284, E376, D377, T372, and E162 (Figure 11d–f) facilitated the correlated cleft-closing of IY-bound ACE (vs. Apo and LL-bound ACE). 

Molecular dynamics simulations of the ACE–lisinopril complex performed by Jalkute et al. [50] indicated that the residues interacting with lisinopril included E376 and D37 in addition to those mentioned previously. It was suggested that these two residues may contribute to the inhibitory effect of lisinopril. In the present study, E376 and D377 contributed to the inhibitory effect of IY by participating in the interaction network.

**Figure 12:**
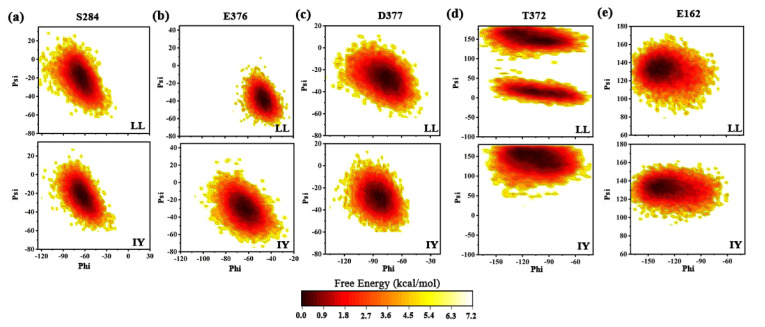
Contour plots of free energies as a function of the backbone angles of φ and ψ for the residues (**a**) E284, (**b**) E376, (**c**) D377, (**d**) T372, and (**e**) E162 of the ACE–LL and ACE–IY complexes. Apo means ligand-free protein, ACE–LL represents the complex system of protein and dipeptide Leu-Leu, ACE–IY stands for complex system of protein and dipeptide Ile-Tyr.

In addition, for the purpose of revealing the energetic characteristics of conformational diversity induced by the LL/IY inhibitor, the free energy landscape was built using variations in the skeletal φ and ψ angles of residues S284, E376, D377, T372, and E162 from GaMD trajectories (Figure 12). Based on Figure 12a, the angle φ and ψ distribution of residue E284 were similar for both the ACE–LL and ACE–IY systems, and the energy minimum corresponded to (−75°, −20°). However, an opposite result was displayed for E376 (Figure 12b). For ACE–LL system, the free energy minimum was located at −50° to −40°, whereas the ACE–LL complex showed a broader distribution. This suggested that the variations in the φ and ψ angles of E376 may provide a contribution to maintaining the stability of hydrogen bonding interaction when ACE is bound to IY. The distribution of energy for D377 and E162 were almost similar for the two complexes (Figure 12c,e). For another aspect, the distribution of T372 was characterized by two minima for ACE–LL (Figure 12d). This may explain the unstable interaction between T372 and E162 in ACE–LL. By comparison, a single minimum was obtained for ACE–IY (Figure 12d). All in all, these results demonstrated that these differences in the angles of φ and ψ of E376 and T372 played a significant role in the conformational diversity for the binding of inhibitors with ACE.

## 4. Conclusions

In this paper, GaMD simulations were used to investigate the molecular mechanisms at the atomic level underlying the large differences in the ACE-inhibitory activity for two dipeptide inhibitors. The results of the trajectory analysis showed that the binding of IY to ACE was more favorable than LL for the formation of a bridge between ACE subdomains, resulting in a closed ligand-binding complex. This suggested that the spatial conformation of the inhibitory peptide was important for its binding to ACE. The effect of binding of two inhibitory peptides on conformational changes of ACE varied widely, mainly in terms of their effects on ACE active pocket volume, in the open and closed states, and on the stability of hinge 2 and hinge 3. We also investigated in-depth the molecular mechanisms underlying these results. This study provided a better understanding of the interaction mechanism of the tACE–peptide complex, which may provide clues for the design of effective peptides against hypertension.

## Figures and Tables

**Table 1:** Average C_α_ RMSD and standard deviations of the whole protein, hinge 1, hinge 2, hinge 3, hinge 4, and the lid during the GaMD simulations in Å.

Regions	Apo	ACE–LL	ACE–IY
Whole	2.45 ± 0.13	2.37 ± 0.08	2.23 ± 0.09
Hinge 1	1.01 ± 0.19	0.72 ± 0.24	0.82 ± 0.24
Hinge 2	1.70 ± 0.35	1.80 ± 0.50	1.50 ± 0.14
Hinge 3	0.84 ± 0.19	2.43 ± 0.35	0.88 ± 0.17
Hinge 4	2.22 ± 0.62	1.63 ± 0.44	1.57 ± 0.33
Lid	2.40 ± 0.20	2.33 ± 0.14	2.23 ± 0.18

## Data Availability

Not applicable.

## Supplementary Materials

The following are available online at https://www.mdpi.com/article/10.3390/foods11030327/s1, Figure S1: Ligand poses of 10 superimposed structures colored by atom type and pose over 1 μs for IY and LL; Figure S2: Time-dependent SASA values for (a) α13, (b) α14, and (c) α15.

## References

[1] Vallumrd S., Oddvang T.K., Severinsson E. (2016). The Evidence of Interdisciplinary Teamwork in the Rehabilitation of Stroke Patients with Aphasia. Open J. Nurs..

[2] Obirikorang Y., Obirikorang C., Acheampong E., Anto E.O., Asiwu R.Y. (2018). Adherence to Lifestyle Modification among Hypertensive Clients: A Descriptive Cross-Sectional Study. Open Access Libr. J..

[3] Wyvratt M.J., Patchett A.A. (1985). Recent developments in the design of angiotensin-converting enzyme inhibitors. Med. Res. Rev..

[4] Natesh R., Schwager S.L., Sturrock E.D., Acharya K.R. (2003). Crystal structure of the human angiotensin-converting enzyme-lisinopril complex. Nature.

[5] Fernandez J.H., Hayashi M.A., Camargo A.C., Neshich G. (2003). Structural basis of the lisinopril-binding specificity in N- and C-domains of human somatic ACE. Biochem. Biophys. Res. Commun..

[6] Brás N., Fernandes P.A., Ramos M.J. (2014). QM/MM Study and MD Simulations on the Hypertension Regulator Angiotensin-Converting Enzyme. ACS Catal..

[7] McDowell S.E., Coleman J.J., Ferner R.E. (2006). Systematic review and meta-analysis of ethnic differences in risks of adverse reactions to drugs used in cardiovascular medicine. BMJ.

[8] Thurman J.M., Schrier R.W. (2003). Comparative effects of angiotensin-converting enzyme inhibitors and angiotensin receptor blockers on blood pressure and the kidney. Am. J. Med..

[9] Atkinson A.B., Morton J.J., Brown J.J., Davies D.L., Fraser R., Kelly P., Leckie B., Lever A.F., Robertson J.I. (2018). Captopril in clinical hypertension. Changes in components of renin-angiotensin system and in body composition in relation to fall in blood pressure with a note on measurement of angiotensin II during converting enzyme inhibition. Br. Heart J..

[10] Redelinghuys P., Nchinda A.T., Sturrock E.D. (2005). Development of domain-selective angiotensin I-converting enzyme inhibitors. Ann. N. Y. Acad. Sci..

[11] Corrons M.A., Liggieri C.S., Trejo S.A., Bruno M.A. (2017). ACE-inhibitory peptides from bovine caseins released with peptidases from Maclura pomifera latex. Food Res. Int..

[12] Ibrahim H.R., Ahmed A.S., Miyata T. (2017). Novel angiotensin-converting enzyme inhibitory peptides from caseins and whey proteins of goat milk. J. Adv. Res..

[13] Grootaert C., Matthijs B., Voorspoels S., Possemiers S., Smagghe G., Van Camp J. (2017). Egg-derived bioactive peptides with ACE-inhibitory properties: A literature update. Food Funct..

[14] Forghani B., Zarei M., Ebrahimpour A., Philip R., Bakar J., Abdul Hamid A., Saari N. (2016). Purification and characterization of angiotensin converting enzyme-inhibitory peptides derived from Stichopus horrens: Stability study against the ACE and inhibition kinetics. J. Funct. Foods.

[15] Ji W., Zhang C., Ji H. (2017). Two Novel Bioactive Peptides from Antarctic Krill with Dual Angiotensin Converting Enzyme and Dipeptidyl Peptidase IV Inhibitory Activities. J. Food Sci..

[16] Lu Z., Xu X., Li D., Sun N., Lin S. (2021). Comprehensive Analysis of Mouse Hippocampal Lysine Acetylome Mediated by Sea Cucumber Peptides Preventing Memory Impairment. J. Agric. Food Chem..

[17] Liu C., Fang L., Min W., Liu J., Li H. (2018). Exploration of the molecular interactions between angiotensin-I-converting enzyme (ACE) and the inhibitory peptides derived from hazelnut (Corylus heterophylla Fisch.). Food Chem..

[18] Moayedi A., Mora L., Aristoy M.C., Safari M., Hashemi M., Toldra F. (2018). Peptidomic analysis of antioxidant and ACE-inhibitory peptides obtained from tomato waste proteins fermented using Bacillus subtilis. Food Chem..

[19] Lin Q., Liao W., Bai J., Wu W., Wu J. (2017). Soy protein-derived ACE-inhibitory peptide LSW (Leu-Ser-Trp) shows anti-inflammatory activity on vascular smooth muscle cells. J. Funct. Foods.

[20] Lee S.Y., Hur S.J. (2017). Antihypertensive peptides from animal products, marine organisms, and plants. Food Chem..

[21] Saleh A.S., Zhang Q., Shen Q. (2016). Recent Research in Antihypertensive Activity of Food Protein-derived Hydrolyzates and Peptides. Crit. Rev. Food Sci. Nutr..

[22] Zheng X., Li D.S., Ding K. (2016). Purification and identification of angiotensin I-converting enzyme inhibitory peptides from fermented walnut residues. Int. J. Food Prop..

[23] Kim H.J., Kang S.G., Jaiswal L., Li J., Choi J.H., Moon S.M., Cho J.Y., Ham K.S. (2016). Identification of four new angiotensin I-converting enzyme inhibitory peptides from fermented anchovy sauce. Appl. Biol. Chem..

[24] Thuanthong M., Gobba C.D., Sirinupong N., Youravong W., Otte J. (2017). Purification and characterization of angiotensin-converting enzyme inhibitory peptides from Nile tilapia (Oreochromis niloticus) skin gelatine produced by an enzymatic membrane reactor. J. Funct. Foods.

[25] Casasnovas R., Limongelli V., Tiwary P., Carloni P., Parrinello M. (2017). Unbinding Kinetics of a p38 MAP Kinase Type II Inhibitor from Metadynamics Simulations. J. Am. Chem. Soc..

[26] Sun H., Li Y., Shen M., Li D., Kang Y., Hou T. (2017). Characterizing Drug–Target Residence Time with Metadynamics: How To Achieve Dissociation Rate Efficiently without Losing Accuracy against Time-Consuming Approaches. J. Chem. Inf. Model..

[27] Tiwary P., Limongelli V., Salvalaglio M., Parrinello M. (2015). Kinetics of protein–ligand unbinding: Predicting pathways, rates, and rate-limiting steps. Proc. Natl. Acad. Sci. USA.

[28] Tiwary P., Mondal J., Morrone J.A., Berne B.J. (2015). The role of water and steric constraints in the kinetics of cavity-ligand unbinding. Proc. Natl. Acad. Sci. USA.

[29] Xu Z., Wu C., Sun-Waterhouse D., Zhao T., Waterhouse G., Zhao M., Su G. (2020). Identification of post-digestion angiotensin-I converting enzyme (ACE) inhibitory peptides from soybean protein Isolate: Their production conditions and in silico molecular docking with ACE. Food Chem..

[30] Feher V.A., McCammon J.A., Miao Y. (2015). Gaussian Accelerated Molecular Dynamics: Unconstrained Enhanced Sampling and Free Energy Calculation. J. Chem. Theory Comput..

[31] Miao Y., McCammon J.A. (2016). Unconstrained Enhanced Sampling for Free Energy Calculations of Biomolecules: A Review. Mol. Simul..

[32] Miao Y., Huang Y.M., Walker R.C., Mccammon J.A., Chang C.E.A. (2018). Ligand Binding Pathways and Conformational Transitions of the HIV Protease. Biochemistry.

[33] Miao Y., Mccammon J.A. (2018). Mechanism of the G-protein mimetic nanobody binding to a muscarinic G-protein-coupled receptor. Proc. Natl. Acad. Sci. USA.

[34] Miao Y., Mccammon J.A. (2016). Graded activation and free energy landscapes of a muscarinic G-protein-coupled receptor. Proc. Natl. Acad. Sci. USA.

[35] Miao Y., Feixas F., Eun C., McCammon J.A. (2015). Accelerated molecular dynamics simulations of protein folding. J. Comput. Chem..

[36] Kappel K., Miao Y., McCammon J.A. (2015). Accelerated molecular dynamics simulations of ligand binding to a muscarinic G-protein-coupled receptor. Q. Rev. Biophys..

[37] Wang J., Miao Y. (2019). Mechanistic Insights into Specific G Protein Interactions with Adenosine Receptors. J. Phys. Chem..

[38] Accelrys Software Inc., Discovery Studio Visualizer v21.1.0. https://discover.3ds.com/discovery-studio-visualizer-download.

[39] Frisch M.J., Trucks G.W., Schlegel H.B., Scuseria G.E., Robb M.A., Cheeseman J.R., Scaknabu G., Baronw V., Petersson G.A., Nakatsuji H. (2009). Gaussian 09, Revision, A.02..

[40] Corradi H.R., Chitapi I., Sewell B.T., Georgiadis D., Dive V., Sturrock E.D., Acharya K.R. (2007). The structure of testis angiotensin-converting enzyme in complex with the C domain-specific inhibitor RXPA380. Biochemistry.

[41] Morris G.M., Huey R., Lindstrom W., Sanner M.F., Belew R.K., Goodsell D.S., Olson A.J. (2009). AutoDock4 and AutoDockTools4: Automated docking with selective receptor flexibility. J. Comput. Chem..

[42] Case D.A., Cerutti D.S., Cheatham T.E., Darden T.A., Duke R.E., Gohlke H., Goetz A.W., Homeyer N., Izadi S., Janowski P. (2016). AMBER.

[43] Maier J.A., Martinez C., Kasavajhala K., Wickstrom L., Hauser K.E., Simmerling C. (2015). ff14SB: Improving the Accuracy of Protein Side Chain and Backbone Parameters from ff99SB. J. Chem. Theory Comput..

[44] Jorgensen W.L.C., Chandrasekhar J., Madura J.D., Impey R.W., Klein M.L. (1983). Comparison of Simple Potential Functions for Simulating Liquid Water. J. Chem. Phys..

[45] Ryckaert J.P., Ciccotti G., Berendsen H. (1977). Numerical integration of the cartesian equations of motion of a system with constraints: Molecular dynamics of n-alkanes. J. Comput. Phys.

[46] Darden T., York D., Pedersen L. (1993). Particle mesh Ewald: An N·log(N) method for Ewald sums in large systems. J. Chem. Phys..

[47] Roe D.R., Cheatham T.E. (2013). PTRAJ and CPPTRAJ: Software for Processing and Analysis of Molecular Dynamics Trajectory Data. J. Chem. Theory Comput..

[48] Ichiye T., Karplus M. (1991). Collective motions in proteins: A covariance analysis of atomic fluctuations in molecular dynamics and normal mode simulations. Proteins.

[49] Miao Y., Sinko W., Pierce L., Bucher D., Walker R.C., McCammon J.A. (2014). Improved Reweighting of Accelerated Molecular Dynamics Simulations for Free Energy Calculation. J. Chem. Theory Comput..

[50] Jalkute C.B., Barage S.H., Dhanavade M.J., Sonawane K.D. (2013). Molecular dynamics simulation and molecular docking studies of Angiotensin converting enzyme with inhibitor lisinopril and amyloid Beta Peptide. Protein. J..

[51] Jiang Z., Zhang H., Bian X., Li J., Li J., Zhang H. (2019). Insight into the binding of ACE-inhibitory peptides to angiotensin-converting enzyme: A molecular simulation. Mol. Simul..

[52] Binkowski T.A., Naghibzadeh S., Jie L. (2003). CASTp: Computed Atlas of Surface Topography of proteins. Nucleic Acids Res..

