# Supplementary material for: Gaussian Accelerated Molecular Dynamics Simulations Investigation on the Mechanism of Angiotensin-Converting Enzyme (ACE) C-Domain Inhibition by Dipeptides
Source: Foods. 2022 Jan 25;11(3):327. doi: 10.3390/foods11030327 (PMC8834632; doi:10.3390/foods11030327)
Supplement: Supplementary file 1 [file foods-11-00327-s001.zip › foods-1490779-supplementary.pdf]

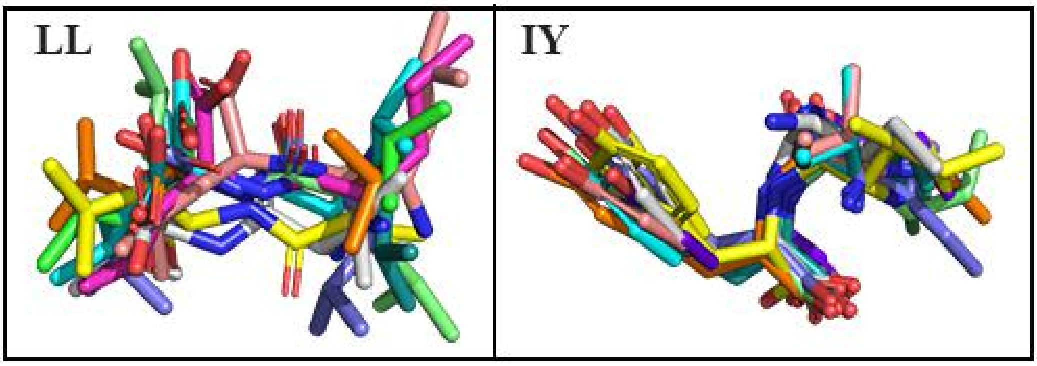

Figure S1. Ligand poses of 10 superimposed structures colored by atom type and pose over 1 μs for IY and LL.

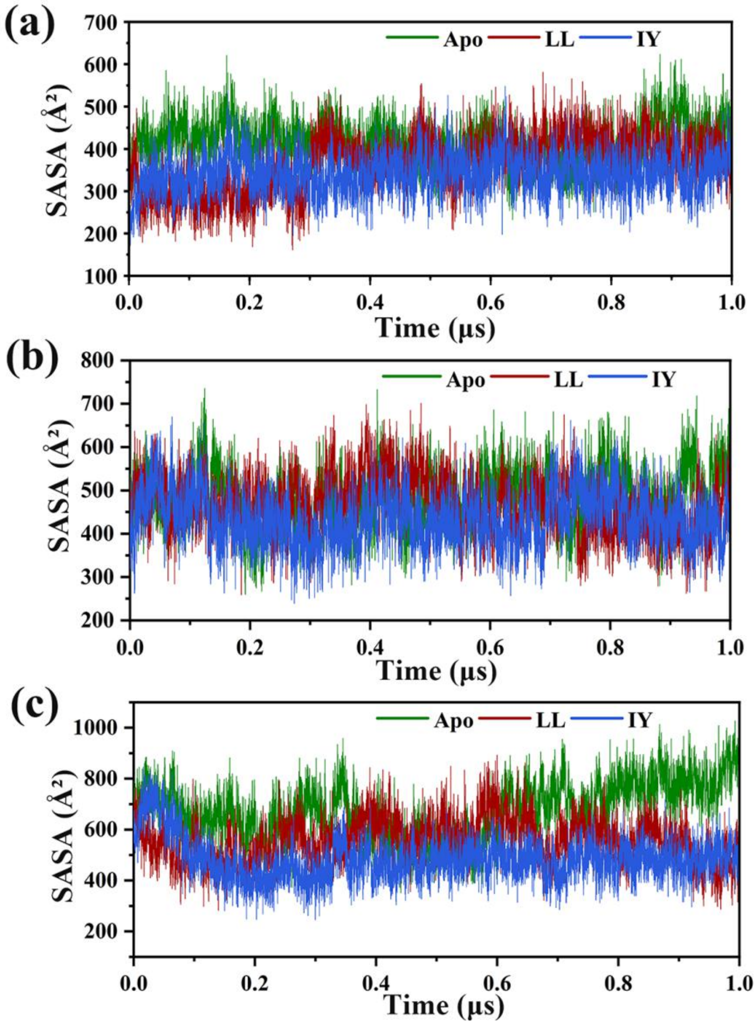

Figure S2. Time dependent SASA values for (a)α13, (b) α14, (c) α15.
